# Effects of antiretroviral therapy in HIV-positive adults on new HIV infections among young women: a systematic review protocol

**DOI:** 10.1186/s13643-019-0982-z

**Published:** 2019-03-05

**Authors:** Trust Chibawara, Lawrence Mbuagbaw, Marcel Kitenge, Peter Nyasulu

**Affiliations:** 1 Division of Epidemiology and Biostatistics, Faculty of Medicine and Health Sciences, Stellenbosch University, Cape Town, South Africa; 2 Department of Health Research Methods, Evidence and Impact, McMaster University, Hamilton, ON Canada; 3 Biostatistics Unit, Father Sean O’Sullivan Research Centre, St. Joseph’s Healthcare, Hamilton, ON Canada; 4 Centre for Development of Best Practices in Health (CDBPH), Yaoundé Central Hospital, Yaoundé, Cameroon; 5 Médecins Sans Frontières, Doctors Without Borders-Rustenburg, Rustenburg, North West South Africa; 6 Division of Epidemiology and Biostatistics, School of Public Health, Faculty of Health Sciences, University of the Witwatersrand, Johannesburg, South Africa

**Keywords:** HIV, AIDS, Antiretroviral treatment, Adolescent girls, Young women

## Abstract

**Background:**

The HIV/AIDS pandemic has struck regions, countries, and populations in different ways. With the introduction of antiretroviral drugs, people living with HIV (PLHIV) have a much better prognosis, even though there are still many new infections in young women. The role of widespread antiretroviral therapy (ART) on the incidence of HIV in young women is unknown.

**Methods:**

We will conduct a comprehensive search of MEDLINE (PubMed), Excerpta Medica database (EMBASE), Scopus, Google Scholar, Cochrane Central Register of Controlled Trials (CENTRAL), World Health Organization’s (WHO’s) library database, Latin American and Caribbean Health Sciences Literature (LILACS), conference abstracts, and gray literature sources to identify any relevant literature. We will include randomized and non-randomized clinical trials and cohort studies in which ART was offered to adults aged 18 and above reporting outcomes in females aged 15 to 24 years. The outcomes of interest are HIV incidence, ART initiation, adherence, retention, and viral load suppression. We will screen titles, abstracts, and the full texts of relevant articles in duplicate. Disagreements will be resolved by consensus. We will extract data on the risk of HIV infection in younger females after the use of ART in the adult population.

**Discussion:**

To our knowledge, this is the first systematic review to look at the impact of ART use among adults on HIV incidence in young women. The results of this review will be used in a modeling study to simulate the effects of using ART as an effective tool to prevent sexual transmission of HIV to young women. Our findings will inform the treatment-as-prevention (TasP) strategy to reduce new HIV infections among young women.

**Systematic review registration:**

The systematic review protocol was registered with the International Prospective Register of Systematic Reviews (PROSPERO), registration number CRD42018099174.

**Electronic supplementary material:**

The online version of this article (10.1186/s13643-019-0982-z) contains supplementary material, which is available to authorized users.

## Background

HIV/AIDS disease burden varies considerably across different regions, countries, and populations. Globally in 2017, there were over 36.9 (95% confidence interval [CI] 31.1–43.9) million people living with HIV of which 19.6 (95% CI 17.5–22.0) million, thus 53% lived in Eastern and Southern Africa [1]. Although Southern Africa is home to less than 1% of the global population, the region has more than a fourth of all HIV infections in the world [1, 2]. On the other hand, HIV incidence has halved from 0.1% of the world population in the mid-1990s to about 0.05% in 2015 [3]. Once thought of as a death sentence on acquisition, HIV is now viewed as a chronic, manageable illness due to the advancement in prevention strategies, education, and research which saw the introduction of the effective antiretroviral therapy (ART).

The randomized controlled trials and implementation studies on HIV treatment which are primarily focused on adults have reported promising reductions in HIV transmission in this population group. Most notable is the HIV Prevention Trials Network 052 (HPTN 052) trial which reported a 96% decrease in HIV transmission and 41% decrease in HIV-related morbidity from early initiation of ART in heterosexual HIV-discordant couples [4]. On the other hand, the ANRS 12249 trial showed no decrease in the HIV incidence as a result of universal test and treat and suggest that if the conditions necessary (linkage in care, ART coverage, ART adherence, retention on treatment) are not met, the benefits of universal test and treat are unlikely to be realized [5]. The ANRS 12249 trial however provided strong evidence on individual benefits and recommended the roll-out of the universal test and treat without any restriction. As a result, scale-up of ART particularly in Southern Africa has contributed to a significant decrease in HIV/AIDS disease burden [6]. Globally, the number of people accessing ART has almost tripled from 7.5 million people in 2010 to 20.9 million in 2017 (61% of whom live in East and Southern Africa) [7].

Young people, on the other hand, are highly vulnerable to new HIV infections. In particular, Anderson et al. [8] and Hayes R et al. [9] highlighted that over half of all new HIV infections worldwide currently occur among young people. In Southern Africa, adolescent girls and young women aged 15–24 contribute to an estimated 74% of all new infections and have up to eight times more infections than their male counterparts [6, 10]. An estimated 2000 new HIV infections are reported to occur among young women and girls every week in South Africa [11]. These high HIV incidence rates in young women have been shown to be largely driven by age-disparate and transactional sexual relationships between young women and older men who recently acquired HIV [12, 13].

Notably “key populations” in turn bears a disproportionate risk of HIV infection compared to the general population [14–18], defined as either men who have sex with men (MSM) or transgender (TG) persons or female sex workers (FSWs) or injecting drug users (IDUs). United Nations Programme on HIV/AIDS (UNAIDS) 2018 [1] estimates just under 50% of global new infections to be attributed to these populations. However, despite well-characterized risks for HIV acquisition and transmission, this group remains a “hard-to-reach” population in part due to stigmatization and criminalization among other reasons [19, 20]. For that reason, we will not be including this population group in our systematic review.

There have been systematic reviews that have focused on interventions aimed at HIV-positive individuals, such as initiation of ART [21, 22], support for ART adherence and retention in care [23–26], linkage to care [27], HIV care [28, 29], and interventions aimed at preventing new infections such as abstinence, be faithful, and condomise (ABC), voluntary male medical circumcision (VMMC), prevention of mother-to-child HIV transmission (PMTCT) [30, 31], microbicides, and pre-/post-exposure prophylaxis (PREP/PEP) [32]. To our knowledge, there is no systematic review that has evaluated the effects of ART intervention studies focused on the adult population on the HIV incidence in young women. In this review, we will measure the effects of ART use on the incidence of HIV in young women.

The data from this review will be used to inform a simulation modeling project, in addition to data from the Eswatini (formerly known as Swaziland)-based implementation study on Early Access to ART for All (EAAA) which evaluated the outcomes associated with offering early ART to all adults with HIV [33].

### Objectives

The objective of this systematic review is to summarize the effects of ART in HIV-positive adults on new HIV infection among young women aged 15 to 24 years.

## Methods and design

This protocol is written in accordance with the Preferred Reporting Items for Systematic Reviews and Meta-Analysis Protocols (PRISMA-P) checklist (see Additional file 1) [34]. This systematic review protocol has been registered and published with the United States National Institute of Health Research (NIHR) Prospective Register of Systematic Reviews (PROSPERO) registration number CRD42018099174.

### Criteria for considering studies for review

#### Types of studies

We will include randomized and non-randomized clinical trials and cohort studies.

#### Type of participants

We will identify studies that include young women aged 15–24 years who are not infected with HIV. The studies should also include HIV-infected adults who are on antiretroviral treatment.

#### Types of interventions/exposures

Adults aged 18 years or more receiving ART: triple drug combination ART given as treatment for HIV/AIDS.

#### Types of outcomes

While the intervention (expanded access to ART) will occur at the population level, the population of interest is the young women aged 15–24 years, and therefore outcomes will only be measured in this target population.

#### Primary outcomes

The incidence of HIV infection in young women aged 15–24 years: the proportion of young women with new infections.

#### Secondary outcomes

The information collected here will be for young women aged 15–24 years. This information will help in synthesizing the information on the conditions necessary to realize the results reported in the primary outcome*.*

ART initiation: the proportion of HIV-positive study participants who started ART.

Retention in care: number of patients who remain connected to medical care as defined by the study authors.

ART adherence: the proportion of participants categorized as adherent to ART based on measures reported by the authors.

Viral load suppression: the proportion of patients with undetectable viral load following ART initiation or as defined by the study authors.

### Exclusion criteria

We will exclude studies that focus on: pediatric populations (defined as infancy, between birth, and 2 years; childhood, 2–12 years and early adolescence, 11–14 years) [35], PREP/PEP interventions, mother-to-child HIV transmission, and key populations, which are defined here as men who have sex with men (MSM) or transgender (TG) persons or female sex workers (FSWs) or injecting drug users (IDUs).

### Search methods for identification of the studies

We will conduct a comprehensive and exhaustive search of published and unpublished literature. We will search the following electronic databases: MEDLINE (detailed search strategy is provided in the Additional file 2), Excerpta Medica Database (EMBASE), Scopus, Google Scholar, Cochrane Central Register of Controlled Trials (CENTRAL), WHO’s library database, and Latin American and Caribbean Health Sciences Literature (LILACS). We will also search conference abstracts from the conferences such as International AIDS Society (IAS) Conference, the International Conference on HIV/AIDS and Sexually Transmitted Infections in Africa (ICASA), and the Conference on Retroviruses and Opportunistic Infections (CROI). We will search for gray literature to identify any relevant unpublished literature, and the identified literature will be appraised through the Authority, Accuracy, Coverage, Objectivity, Date, and Significance (AACODS) checklist [36].

We will also check the reference lists of all included studies and relevant systematic reviews to identify additional studies missed from the original electronic searches.

We will restrict our search to articles published in English and those published from 1996 (start of the triple-drug combination ART era).

### Selection of studies

The results of the search will be uploaded in Covidence [37], a web-based online platform which facilitates screening and selection of articles. The screening will be carried out in two stages using pre-specified screening criteria. First, titles and abstract screening will be done by two reviewers (TC, MK) working independently.

Full-text versions of the relevant studies will then be downloaded and screened for eligibility by two reviewers (TC, MK) working independently. We will extract data from the selected studies onto a pre-tested Microsoft Excel spreadsheet. If any disagreements occur, these will be resolved by discussion and if disagreements persist, it will be resolved by arbitration from a third reviewer (LM or PN).

### Data extraction, management, and analysis

Data from the full-text articles will be extracted by two independent authors (TC, MK) using a standardized extraction form. Any discrepancy will be resolved by consensus or with referral to a third author if disagreement persists. The data extraction form to be used to extract relevant information from the eligible studies has been developed to include four main heading: bibliometric information, participant’s demographics, reported outcomes, and article research variables. A detailed description of the extraction form is provided in Additional file 3. Any changes to be made during the extraction process will be reported in the final manuscript.

In the event that there is missing information, we need clarification about study conduct or the studies have relevant data that is not reported in the published manuscript, we will contact the authors for additional information which may include de-identified individual patient data.

Risk of bias in randomized studies will be assessed using the Cochrane “risk of bias” tool [38], and the risk of bias for cohort studies will be assessed using the risk of bias in non-randomized studies of interventions (ROBINS-I) [39].

Though we do not anticipate to having sufficient data to perform a meta-analysis, we state here the possibility that if the identified studies are relatively homogeneous in terms of methodology and outcomes, meta-analyses of the data will be performed. Funnel-plots and Egger’s test will be used to determine publication bias. Sufficiently similar data will be pooled using the inverse variance approach to accommodate crude and adjusted odds ratios where possible. Given the purpose of this review, i.e., to collect data to inform a simulation model, no subgroup analyses are planned.

Statistical heterogeneity will be quantified using the *I*^2^ statistic, with an *I*^2^ statistic of 75–100 considered high [40]. A random effects model will be used, and we will report prediction intervals (PI) as opposed to CIs for better appreciation of uncertainty around effect estimate [41]. If clinical heterogeneity precludes a meta-analysis, a narrative synthesis will be performed. All analysis will be performed using R software [42].

The quality of evidence will be assessed using the Grading of Recommendations Assessment Development and Evaluation (GRADE) approach [43]. An overall GRADE certainty of evidence (high, moderate, low, very low) will be based on an appraisal of the body of evidence for items such as the risk of bias, imprecision, indirectness, and publication bias. Any disagreements will be recorded and resolved by consensus.

## Discussion

Preventing new HIV infection is a critical component of the global response to the epidemic [44]. The body of evidence supporting the efficacy of TasP is expanding quickly. However, most of the studies are still focused on the adult population [45] with limited data on outcomes in young women.

To bridge this gap of evidence, the estimates from this review will inform an individual-based heterosexual HIV transmission simulation modeling, which will also include data from an Eswatini-based implementation study on Early Access to ART for All (EAAA) [33]. The EAAA study aims to evaluate the feasibility, acceptability, clinical outcomes, affordability, and scalability of offering early ART to all adults with HIV aged 18 years or more in the government-managed health system.

The EAAA study will provide clinical, biological, and behavioral data to ensure that the model mimics the real-world HIV transmission and incidence among young women.

Evidence from this systematic review will provide valuable insights to the modeling arm of the project by synthesizing evidence from studies that measure the effects of ART among HIV-positive adults on HIV infection among young women in diverse settings.

This protocol has some limitations. It may be challenging to find studies with data on women aged less than 18 years because the legal age of consent is 18 years in most countries. Due to limited translation resources, we are only able to review articles published in English—this could result in articles that are published in other languages being excluded.

The strengths of this project lie in its novelty. This is the first systematic review to look at the impact of ART use among adults on HIV infection in young women. Our robust search strategy will ensure a comprehensive body of evidence.

We anticipate that the review will provide valuable information to inform the WHO 90-90-90 targets (diagnose 90% of all HIV-positive individuals and provide antiretroviral treatment to 90% of those diagnosed with HIV and for those on treatment, 90% will achieve viral suppression by 2020) [46]. We speculate that our model, which models the effect of providing ART to 90% of the people with HIV, will show a reduction in new HIV infection among young women in support of and reinforcing the need to adopt the TasP strategy to curb new HIV infections among young women. Any changes made to the protocol will be reported in the final manuscript.

### Ethics and dissemination

The study does not require ethical approval because we will use already published data extracted from manuscripts. The results of this systematic review will be disseminated in peer-reviewed journals and conference presentations.

## Additional files

Additional file 1: PRISMA-P 2015 checklist. (DOCX 30 kb)

Additional file 2: Draft search strategy—MEDLINE (PubMed). (DOCX 15 kb)

Additional file 3: Data extraction form field description. (XLSX 11 kb)

## Abbreviations

ABC: Abstinence, be faithful, and condomise
AIDS: Acquired immune deficiency syndrome
AIDS: Acquired immunodeficiency syndrome
ANRS: Agence Nationale de Recherch sur le Sida
ART: Antiretroviral therapy
CDBPH: Centre for Development of Best Practices in Health
CENTRAL: Cochrane Central Register of Controlled Trials
CI: Confidence interval
CROI: Conference on Retroviruses and Opportunistic Infections
EAAA: Early Access to ART for All
EMBASE: Excerpta Medica database
FSWs: Female sex workers
GRADE: Grading of Recommendations Assessment Development and Evaluation
HIV: Human immunodeficiency virus
HPTN: HIV Prevention Trials Network
IAS: International AIDS Society
ICASA: International Conference on HIV/AIDS and Sexually Transmitted Infections in Africa
IDUs: Injecting drug users
LILACS: Latin American and Caribbean Health Sciences Literature
MEDLINE: Medical Literature Analysis and Retrieval System Online
MSM: Men who have sex with men
NIHR: National Institute of Health Research
PEP: Post-exposure prophylaxis
PI: Predictive interval
PLHIV: People living with HIV
PMTC: Prevention of mother-to-child HIV transmission
PREP: Pre-exposure prophylaxis
PRISMA-P: Preferred Reporting Items for Systematic Reviews and Meta-Analysis Protocols
PROSPERO: Prospective Register of Systematic Reviews
PubMed: Public/Publisher MEDLINE
ROBINS-I: Risk of Bias in Non-randomized Studies of Interventions
TasP: Treatment-as-prevention
TG: Transgender
UNAIDS: United Nations Programme on HIV/AIDS
VMMC: Voluntary male medical circumcision
WHO: World Health Organization
