# Supplementary material for: Effects of antiretroviral therapy in HIV-positive adults on new HIV infections among young women: a systematic review protocol
Source: Syst Rev. 2019 Mar 5;8:68. doi: 10.1186/s13643-019-0982-z (PMC6402083; doi:10.1186/s13643-019-0982-z)
Supplement: Supplementary file 2 — Draft search strategy—MEDLINE (PubMed). (DOCX 15 kb) [file 13643_2019_982_MOESM2_ESM.docx]

**Additional file 1: Draft Search Strategy – Medline (PubMed)**

#1 Search ((HIV OR hiv-1 OR hiv-2* OR hiv1 OR hiv2 OR hiv infect* OR human immunodeficiency virus

OR human immune deficiency virus OR human immuno-deficiency virus OR human immune-

deficiency virus OR ((human immun*) AND (deficiency virus)) OR acquired immunodeficiency

syndromes OR acquired immune deficiency syndrome OR acquired immuno-deficiency syndrome OR

acquired immune-deficiency syndrome OR ((acquired immun*) AND (deficiency syndrome)) OR

HIV/AIDS))

#2 Search ((HIV infections [MeSH] OR HIV [MeSH]))

#3 Search (#1 OR #2)

#4 Search ((Antiretroviral* OR ((anti) AND (retroviral*)) OR ARV* OR ART OR “antiretroviral therapy”

OR HAART OR ((highly) AND (active) AND (antiretroviral*) AND (therap*)) OR ((anti) AND (hiv)) OR

((anti) AND (acquired immunodeficiency)) OR ((anti) AND (acquired immuno-deficiency)) OR ((anti)

AND (acquired immune-deficiency)) OR ((anti) AND (acquired immun*) AND (deficienc*))))

#5 Search ((antiretroviral agents [Mesh] OR antiretroviral therapy, highly active [Mesh]))

#6 Search (#4 OR #5)

#7 #3 AND #6

#8 “mixed hiv” OR mixed-status OR serodiscordant OR sero-discordant OR serostatus OR sero-

conversion OR seroconversion

#9 #3 AND #6 AND #8
